# Suppression of Luminance Contrast Sensitivity by Weak Color Presentation

**DOI:** 10.3389/fnins.2021.668116

**Published:** 2021-06-28

**Authors:** Ippei Negishi, Keizo Shinomori

**Affiliations:** ^1^School of Information, Kochi University of Technology, Kami, Japan; ^2^Department of Media Informatics, College of Informatics and Human Communication, Kanazawa Institute of Technology, Hakusan, Japan; ^3^Vision and Affective Science Integrated Laboratory, Research Institute, Kochi University of Technology, Kami, Japan

**Keywords:** luminance-color interaction, fMRI, visual psychophysics, luminance contrast, visual attention

## Abstract

The results of psychophysical studies suggest that color in a visual scene affects luminance contrast perception. In our brain imaging studies we have found evidence of an effect of chromatic information on luminance information. The dependency of saturation on brain activity in the visual cortices was measured by functional magnetic resonance imaging (fMRI) while the subjects were observing visual stimuli consisting of colored patches of various hues manipulated in saturation (Chroma value in the Munsell color system) on an achromatic background. The results indicate that the patches suppressed luminance driven brain activity. Furthermore, the suppression was stronger rather than weaker for patches with lower saturation colors, although suppression was absent when gray patches were presented instead of colored patches. We also measured brain activity while the subjects observed only the patches (on a uniformly black background) and confirmed that the colored patches alone did not give rise to differences in brain activity for different Chroma values. The chromatic information affects the luminance information in V1, since the effect was observed in early visual cortices (V2 and V3) and the ventral pathway (hV4), as well as in the dorsal pathway (V3A/B). In addition, we conducted a psychophysical experiment in which the ability to discriminate luminance contrast on a grating was measured. Discrimination was worse when weak (less saturated) colored patches were attached to the grating than when strong (saturated) colored patches or achromatic patches were attached. The results of both the fMRI and psychophysical experiments were consistent in that the effects of color were greater in the conditions with low saturation colors.

## Introduction

Numerous studies, including some by the present authors, have the investigated the interaction between chromatic and luminance information in the human visual system. The most general example of such an interaction is the change in color perception resulting from changes in the luminance of the surrounding background, referred to as blackness induction (Shinomori et al., 1994, 1997). Some psychophysical studies have reported interactions between chromatic and luminance information on luminance (Switkes et al., 1988; Kingdom, 2003; Kingdom and Kasrai, 2006; Miquilini et al., 2017; Sousa et al., 2020), orientation (Clifford et al., 2003; Kingdom et al., 2010) and object shape (Clery et al., 2013) perception, while others have provided evidence that there is no effect (Victor et al., 1998; Xiao and Wade, 2010). Because of differences in the visual conditions and tasks used in those studies, it is difficult to conclude whether this interaction exists or not. Switkes et al. (1988) reported experimental evidence of an interaction between chromatic and luminance information on the basis of suppression of luminance information by chromatic information. Subsequent articles confirmed those results (Kingdom, 2003; Kingdom and Kasrai, 2006; Kingdom et al., 2010; Miquilini et al., 2017; Sousa et al., 2020); thus, we expected that some required conditions for suppression would exist. In former studies the saturation of color stimuli had not been considered, so in this study we specifically manipulated the saturation of the color in visual stimuli. In these studies, chromatic and luminance stimuli were presented at a spatially identical or proximate position, so the subjects observed the combined signal of color and luminance, and most of the subjects could not perceive chromatic and luminance information independently. Therefore, we presented chromatic stimuli in the surround of the visual field because we tried to measure the effect of chromatic information on luminance information rather than the antagonistic interaction between luminance and chromatic information.

There are two principal pathways in the human visual cortices (Goodale and Milner, 1992). Many studies have demonstrated that the visual cortices in the ventral pathway respond to chromatic stimuli (Mullen et al., 2007; Wade et al., 2008) and those in the ventral pathway processes features of objects, thus the ventral pathway is often referred to as the “what” pathway. With respect to chromatic signals the ventral pathway has been thought of as the main processing site. The dorsal pathway mostly processes information about environmental space and three-dimensional perception, and hence is often referred to as the “where” pathway. The information processed in the dorsal pathway seems to be mediated primarily by luminance signals. However, the results of some previous studies indicate that chromatic information influences the dorsal pathway under specific conditions, such as color defined motion stimuli (Maunsell, 1992; Tootell et al., 1995; Wandell et al., 1999), suggesting that the dorsal and ventral pathways are connected via the ventral occipital fasciculus (Yeatman et al., 2014; Xing et al., 2015; Takemura et al., 2016). This would suggest that luminance information receives the effect of chromatic information before the divergence of the dorsal and ventral pathways. Primary or early visual cortices, especially human V1, are known to respond to chromatic stimuli (Engel et al., 1997; Mullen et al., 2007); luminance-selective, color-selective and both luminance- and color-selective neurons were found in macaque V1 (Livingstone and Hubel, 1984; Johnson et al., 2001), human visual cortices (V1, V2, and V3), the ventral pathways (hV4), and the dorsal pathways (V3A/B) in observing visual stimuli consisting of luminance and chromatic components (Bartels and Zeki, 2000; Brouwer and Heeger, 2009). Through this experiment, we aimed to clarify the stimulus conditions for the interaction between luminance and chromatic information, and to identify the visual cortex where this interaction occurs.

It is broadly accepted and supported by the results of numerous psychophysical (Brefczynski and DeYoe, 1999; Somers et al., 1999; Turatto and Galfano, 2000; Morrone et al., 2002; Fuller and Carrasco, 2006) and physiological (Bannert and Bartels, 2013) studies that human visual perception is influenced by attention. The amplitude of blood-oxygen-level dependent (BOLD) responses in early human visual cortices is also modulated by subject attention (Brefczynski and DeYoe, 1999; Somers et al., 1999; Turatto and Galfano, 2000; Morrone et al., 2002; Fuller and Carrasco, 2006). Additionally, brain activity patterns can receive the effect of the attention from higher-level cortices (Bannert and Bartels, 2013). As we thought attention might also affect the interaction between luminance and color (although the clear evidence of this point has not been shown), we measured the effect of the chromatic information when subjects were not required to attend to some specific feature in their field of vision, as the tasks in previous experiments (Switkes et al., 1988) did not require active attention.

We firstly conducted a physiological experiment to measure brain activity using fMRI. However, since we obtained unexpected results, we subsequently performed a psychophysical experiment to confirm that the fMRI results were in accordance with psychophysical results obtained for similar visual stimuli (see “Psychophysical Experiment” section for details). The results of the fMRI experiment provided evidence of the suppression of luminance information by chromatic information, and confirmed that the colored patches themselves did not give rise to differences in brain activity for different Chroma values. Interestingly, when the saturation of the patches was lower there was an interaction such that the suppression was stronger rather than weaker. The results of the fMRI experiment were supported by the results of the psychophysical experiment measuring luminance contrast discrimination ability.

## fmri Experiment

### Methods

#### Subjects

Thirteen subjects (twelve male and one female, mean age: 22.7 ± 2.1 years) participated in the fMRI experiment. All subjects had normal or corrected to normal visual acuity better than 1.4 min of visual angle. Their color vision was classified as color normal by traditional color vision tests: Ishihara-plate (International 38 plates edition); Standard Pseudo-isochromatic Plates (SPP), and Panel D-15.

#### Visual Stimuli

Visual stimuli were presented on a rear projection screen using an LCD projector (DLA-X70R-B, Victor). A special visual stimulus presentation system (ViSaGe, Cambridge Research Systems, Inc.) was used to generate the visual stimuli. The subjects observed the screen by means of a mirror attached to the head coil. The resolution of the projector was 1,280 (horizontal) × 1,024 (vertical) pixels and the stimuli were presented in 30-bit color mode. The angular size of the visual stimuli was 31.5° × 25.2° at a 69 cm viewing distance. Chromaticity coordinates and luminance of all colors in the stimuli were controlled and calibrated on the presentation screen, using the special visual stimulation presentation system and its Gamma and chromaticity coordinates calibration data. The measurements were performed directly on the screen by means of a colorimeter (CS-200, Konica-Minolta, Inc.) and spectral radiometer (CS-1000, Konica-Minolta, Inc.) confirming that the screen presentation error was less than 0.005 for CIE (Commission Internationale de l’Éclairage) 1931 xy chromaticity coordinates, and less than 5% for luminance.

**FIGURE 1:**
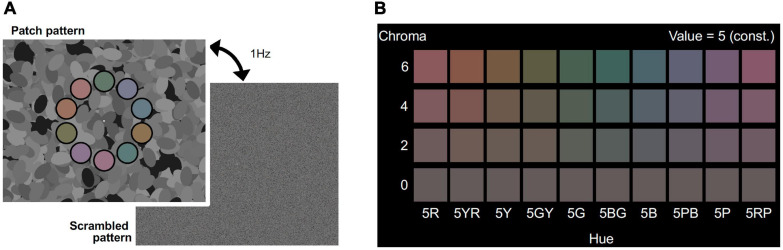
**(A)** Visual stimuli for the fMRI experiment. Visual stimuli alternating at 1 Hz between the patch pattern (top) and the scrambled pattern (bottom) was presented in the stimulation phase of the fMRI experiment. Colors of the 10 patches had the same Chroma (/6, /4, /2, or /0) and Value (5/) of Munsell color system. Achromatic ellipses in the background had one of eight luminance levels (from 0.3 to 30.4 cd/m^2^). Scrambled patterns were generated by randomly shuffling the coordinates of pixels in the patch patterns. The fixation point was set at the center of the screen. **(B)** Set of colors for patches in the four Chroma conditions. Chroma value of each visual stimulus was either /6 (Top raw), /4 (second raw), /2 (third raw), or /0 (bottom raw). All patches had the same gray color in Chroma /0 condition.

Visual stimuli consisted of patch and scrambled patterns, as shown in Figure 1A. The patch pattern consisted of ten colored patches, the centers of which were placed on a 12.4° diameter circle with equal arc angle (36.0°) on an achromatic background (top panel of Figure 1A). The diameter of each patch was 3.0° and each patch had a 15 min. wide black fringe. The same background pattern, consisting of 600 achromatic ellipses of fixed size (3.3° × 1.8°) with randomized orientation and position, was used for all stimuli. In this manner, we could present luminance edges to evoke brain activity while avoiding a possible anisotropic effect (Loffler and Orbach, 2001; Krukowski and Stone, 2005). The luminance of the ellipses was assigned randomly as one of eight levels from 0.23 to 30.24 cd/m^2^ and the mean luminance was 15.4 cd/m^2^. The multiple levels of luminance could make more edges than simple two-leveled luminance, and reduce luminance induction effects (Shinomori et al., 1994, 1997). A white fixation rectangle was presented at the center of the screen. Each patch had one of ten different color hues to avoid hue specific activations (Engel et al., 1997; Shinomori and Werner, 2012; Kuriki et al., 2015) and the colors are presented in Figure 1B. The hues were calibrated to 5R, 5YR, 5Y, 5GY, 5G, 5BG, 5B, 5PB, 5P, and 5RP in the Munsell color system under a D65 lightning condition. The Munsell Value (which corresponds to lightness) of the patches was fixed to 5/ (approximately 16.0 cd/m^2^). The Chroma value (which corresponds to saturation) was manipulated as an experimental parameter and was set to one of /6, /4, /2, and /0. The Chroma /0 condition represents achromatic patches, meaning the color of the ten patches was an identical gray color, so no effect of chromatic information was expected in that condition. The subjects could distinguish each hue in the magnetic resonance imaging (MRI) scanner in the /6, /4, and /2 Chroma conditions. The position of each patch was randomly chosen from among three conditions: the uppermost of the patches was in either the 12 o’clock position, rotated 12° clockwise, or rotated 12° counterclockwise. The scrambled pattern was generated by randomizing the location of the pixels. All pixels on the screen, including background pixels (but excluding the white fixation rectangle), were randomized. The fixation point was set at the center of the screen.

In the control experiment described later, the achromatic patterns (ellipses) were removed from the background, so that the background was uniformly black (0.05 cd/m^2^). In the scrambled pattern in the control experiment, only pixels in the locations of the patches were pseudorandomized and the rest of the screen was kept continuously black.

#### Procedure

A block design was employed for the experimental sequence. One block consisted of stimulation and resting phases, and each phase lasted 15 s. In the stimulation phase the patch and scrambled patterns were altered at 1 Hz to sustain the activation of the visual cortex (Figure 1A). The arrangements of the hues of the ten patches were consistent in each block and randomized among blocks. The subjects had no task in the stimulation phase; they were only required to observe the visual stimuli. The purpose of the resting phase was to reset brain activity.

An easy cognitive task, detecting a change of a central target between “+” and “o” occurring at 50% probability, was performed in the first 5 s of the resting phase so as to sustain and confirm awareness of the subject. If the subject erred (including no response) more than three times in one run, the fMRI data in that run was not included in further analyses. The correct answer rate of the cognitive task was 98.7% (SEM: 0.4%). The last 10 s of the resting phase was completely rest time and nothing was presented on the screen.

We set each block as a pair of one stimulation and one resting phase. One run consisted of one dummy block and twelve subsequent blocks performed sequentially, and each Chroma condition (/6, /4, /2, and /0) was presented three times in pseudorandom order. Each subject performed 18 runs and the subjects could rest between runs for an unlimited period of time in or out of the scanner. The experiments were conducted over several days.

#### MRI Parameters

MRI data were collected on a Siemens 3T scanner (Verio) and a 32 ch head coil. The subjects who needed visual acuity correction wore goggles with appropriate corrective lenses. Structural MRI images (T1-weighted images) were collected by an MPRAGE sequence with TR = 2,250 ms, TE = 3.85 ms, FA = 9°, and 1.0 mm × 1.0 mm × 1.0 mm voxel size. Functional images were collected by an EPI sequence with TR = 2,500 ms, TE = 30 ms, FA = 80°, and 3.0 mm × 3.0 mm × 3.0 mm voxel size. The images consisted of 38 slices (64 × 64 voxel) with 0.45 mm gaps.

#### MRI Data Processing

We used BrainVoyager QX (ver. 2.8) for the processing and analysis of the MRI images. We realigned functional images to remove head movement during experiments and aligned them to the structural image of each subject with a six-parameter affine transformation. We applied a temporal high pass filter to functional images to reduce noise. No spatial filters were applied to images because spatial resolution was considered more important than the S/N ratio, and the number of blocks was sufficient to secure reasonable S/N ratios. BOLD responses were averaged for each Chroma condition and each visual cortex. Time courses of the BOLD response obtained from 54 blocks (three blocks per run × 18 runs) were averaged (excluding the data of one run that was removed for two subjects due to a low awareness level as defined by attention to the cognitive task).

**FIGURE 2:**
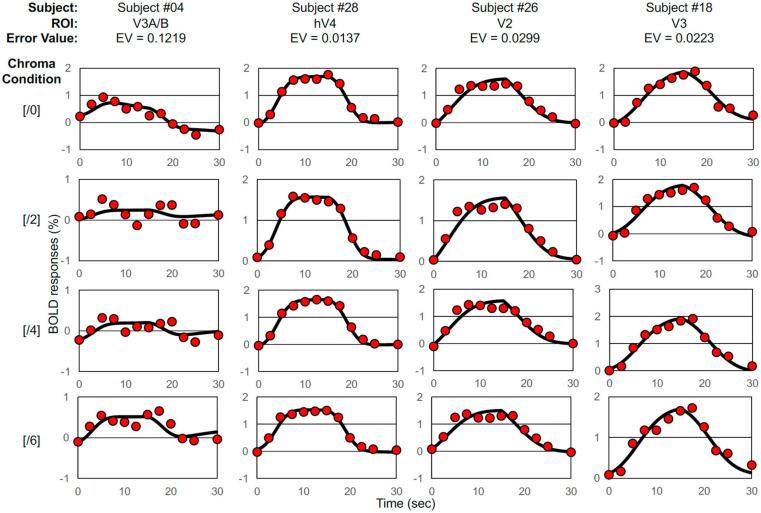
Examples of BOLD signal data and model fits. Selected BOLD signal data (denoted by red circles) and model fittings generated by the two-Gaussian pulse model (Eq. 1, solid curves) under Chroma conditions from /0 (top raw) to /6 (bottom raw), the worst-case fitting (EV = 0.1219) (first column), the best-case fitting (EV = 0.0137) (second column), the case of fitting where EV was around the average (EV = 0.0299) (third column), and the case of fitting where EV was the median (EV = 0.0223) (forth column).

We defined five ROIs using the traveling wave method (Wandell et al., 2007) and calculated the change in the BOLD response in those ROIs (Figure 2). We selected a two-Gaussian hemodynamic response function (HRF) model (Rajapakse et al., 1998) and applied it to the discrete time course of each BOLD response for each visual cortex and each subject. The model response (***ModelR*_*ch*_**) at time ***t*_*n*_** is expressed by Eq. 1.

$$\begin{array}{c} {\mathrm{ModelR}}_{\mathrm{ch}}\left(t_{n}\right)=\int_{t_{n}-15}^{t_{n}}[a_{1,\mathrm{Ch}}H\left(\tau \right)\times \text{exp}\left\{\frac{-{\left(\tau -T_{1}\right)}^{2}}{2\,\alpha_{1}^{2}}\right\} \end{array}$$

$$\begin{array}{c} +a_{2,C\,h}H\left(\tau \right)\times \text{exp}\left\{\frac{-{\left(\tau -T_{2}\right)}^{2}}{2\,\alpha_{2}^{2}}\right\}]d\tau \end{array}$$

(1) $$\begin{array}{c} +\left(b_{C\,h}\,t_{n}+d_{C\,h}\right) \end{array}$$

Where ***a*_1_** (positive value) and ***a*_2_** (negative value) are the intensity parameters of two Gaussian pulses; ***T*_1_**, ***T*_2_**, ***α_1_***, and ***α_2_*** are the four temporal parameters of the pulses; ***H(τ)*** is the Heaviside step function; ***b_*ch*_t_*n*_* + *d_*ch*_*** is the linear component, and the discrete time ***t*_*n*_** (***n*** = 0–12) is the series time in seconds (***t*_*n*_** = 0.0, 2.5, 5.0, 7.5, 10.0, 12.5, 15.0, 17.5, 20.0, 22.5, 25.0, 27.5, and 30.0). In the model fit, ***a*_1_**, ***a*_2_**, ***b***, and ***d*** were varied for each data fit to minimize Error Value (***EV***) defined by Eq. 2 where the four temporal parameters (***T*_1_**, ***T*_2_**, ***α_1_***, and ***α_2_***) were identical for each subject and each cortex through all four Chroma conditions (***Ch*** = /0, /2, /4, and /6):

(2) $$E\,V=\sum_{C\,h=0,/2,/4,/6}\left[\sum_{n=0}^{12}{\left[\frac{\left\{R_{C\,h}\,\left(t_{n}\right)-M\,o\,d\,e\,l\,R_{C\,h}\,\left(t_{n}\right)\right\}}{M\,o\,d\,e\,l\,R_{C\,h}\,\left(t_{n}\right)}\right]}^{2}\right]$$

**FIGURE 3:**
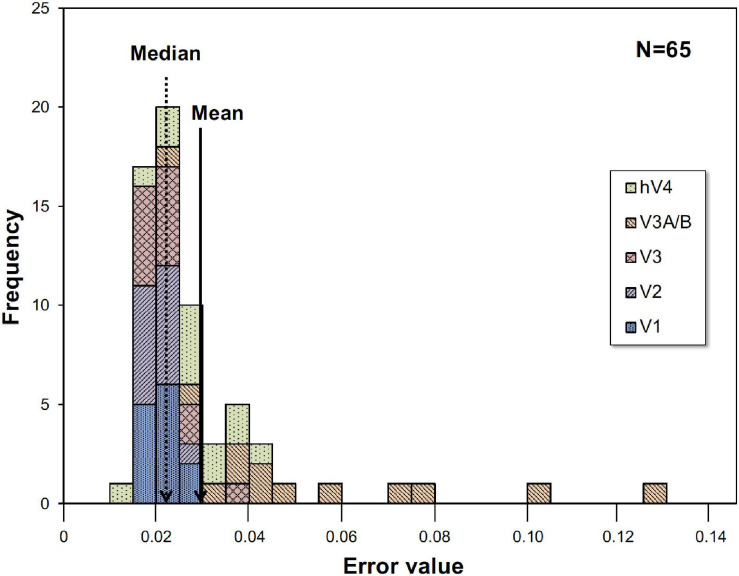
Histogram of error value (EV). Histogram for all thirteen subjects and five visual cortices. EVs were large in V3A/B for some subjects.

Where ***R*_*Ch*_** is the measured BOLD response, ***ModelR*_*Ch*_** is the best-fit model response of Eq. 1, and ***t*_*n*_** is the discrete time. We employed the peak point of the fitted curve as the response (% signal change) under each condition, interpreted as responses to visual stimuli. The best, worst and intermediate (around the average and the median) examples of application are shown in Figure 2. The left and right hemisphere responses were combined for analysis. Figure 3 is a histogram of ***EV*** in all fittings for each subject and each visual cortex (total ***N*** = 65). The distribution of the error in the model fits was small except for the V3A/B data of some subjects. ***EV***s were large in V3A/B for some subjects because the BOLD response of V3A/B was the weakest among the visual fields.

#### Visual Cortex Segmentation

We measured retinotopic mappings using the traveling wave method (Wandell et al., 2007), defining V1, V2, V3, V3A/B, and V4 separately for each subject. We attempted to keep the region of lateral occipital area 1 (LO1) separated so as to clarify the border with V3 and V3A/B. However, since the anterior border of LO1 was not clear, we did not include LO1 in the BOLD signal analysis. We used a flickering checkerboard pattern (11.9° × 11.9° of visual angle) of minimum black (0.05 cd/m^2^) and maximum white (360.3 cd/m^2^) on the screen, a wedge-shaped aperture moving at a polar angle (45°), and a ring shape of eccentricity from 0 to 11.9°. Cycle time was 24 s. Functional images were collected by means of EPI sequence with TR = 2,000 ms, TE = 30 ms, FA = 80°, and 3.0 mm × 3.0 mm × 3.0 mm voxel size. In addition to BrainVoyager QX (ver. 2.8), we used FreeSurfer (ver. 5.3.0) for segmentation of white and gray matter. A typical example of visual field segmentation is displayed in Supplementary Figure 1.

### Results

**FIGURE 4:**
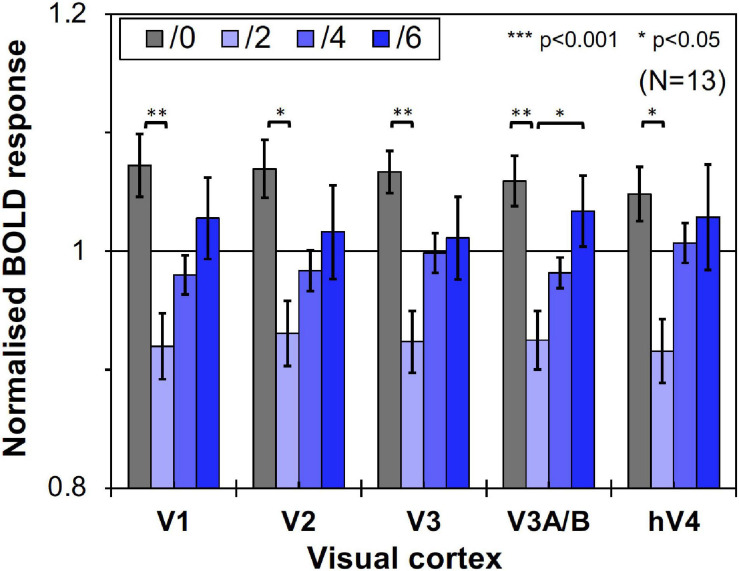
Brain activity of visual cortices under the four Chroma conditions (/0, /2, /4, and /6). The vertical axis units are percent amount of signal change defined as incremental rate of BOLD response subtracted from the mean of the incremental rate under all four Chroma conditions in the stimulation phase. Error bars denote SEM. Asterisks denote statistical significance by Tukey’s honestly significant difference (HSD) test (**p < 0.01; *p < 0.05).

We compared brain activity measured while the subjects were observing alternating visual stimuli (Figure 1A) in the four Chroma conditions of the patches. Brain activity of each subject in each condition was expressed by the normalized BOLD response; each BOLD response was normalized by the average of all four Chroma conditions in all runs (216 blocks). The means of the averaged BOLD response (% signal change) under all four Chroma conditions for all subjects were 3.14% (V1), 2.22% (V2), 1.80% (V3), 0.706% (V3A/B), and 1.44% (hV4). We compared the normalized BOLD response between the Chroma conditions (Figure 4). Brain activity in the Chroma /0 condition was the largest in all identified visual cortices, and those in the Chroma /6, /4, and /2 conditions showed a decreasing order of strength (from /6 to /4 to /2) in all visual cortices analyzed (V1, V2, V3, V3A/B, and hV4). A one-way repeated measures (within subjects) analysis of variance (ANOVA) was performed for the data before normalization, and showed a significant main effect of the Chroma condition in all measured visual cortices (Table 1). Activity in the Chroma /2 condition was significantly smaller than that in the Chroma /0 condition, as shown by Tukey’s HSD test (V1: *p* = 0.007; V2: *p* = 0.024; V3: *p* = 0.006; V3A/B: *p* = 0.004; and hV4: *p* = 0.045) for all cortices, and in Chroma /6 condition for V3A/B (*p* = 0.044). These results suggest that the Chroma of the patches modulated brain activity of the visual cortices, but the order of Chroma values (/0, /2, /4, and /6) showed no correlation with the order of the change in the signal strength of brain activity.

**TABLE 1:** ANOVA results of fMRI experiments for each visual cortex.

		**Main experiment**				**Control experiment**			
**Source**	**D*f***	**Sum Sq**	**Mean Sq**	***F* value**	***p***	**Sum Sq**	**Mean Sq**	***F* value**	***p***
V1									
Chroma condition	3	0.167	0.056	4.392	0.001**	0.007	0.002	0.064	0.979
Subject	12	22.547	1.879			21.141	1.762		
Residuals	36	0.457	0.013			1.316	0.037		
V2									
Chroma condition	3	0.132	0.044	3.154	0.037*	0.020	0.007	0.177	0.912
Subject	12	13.325	1.353			13.894	1.158		
Residuals	36	0.504	0.014			1.389	0.039		
V3									
Chroma condition	3	0.136	0.045	4.166	0.012*	0.071	0.024	0.896	0.452
Subject	12	7.507	0.626			6.729	0.561		
Residuals	36	0.390	0.011			0.944	0.026		
V3A/B									
Chroma condition	3	0.140	0.047	5.113	0.005**	0.082	0.027	1.179	0.331
Subject	12	3.771	0.314			12.442	1.037		
Residuals	36	0.329	0.009			0.836	0.023		
hV4									
Chroma condition	3	0.134	0.045	2.933	0.046*	0.236	0.079	1.783	0.168
Subject	12	9.103	0.759			10.982	0.915		
Residuals	36	0.548	0.015			1.587	0.044		
V1 (inside the patch)									
Chroma condition	3	0.293	0.097	5.009	0.005**	0.085	0.028	0.767	0.520
Subject	12	14.950	1.246			9.791	0.816		
Residuals	36	0.702	0.020			1.333	0.037		
V1 (on the patch)									
Chroma condition	3	0.160	0.054	5.521	0.003**	0.061	0.020	0.564	0.642
Subject	12	39.230	3.269			28.136	2.345		
Residuals	36	0.350	0.010			1.246	0.036		

**p < 0.001; *p < 0.05.

We thought that the brain activity observed in the experiment should correspond to that under achromatic pattern backgrounds, since from the viewpoint of retinotopy, the brain regions corresponding to the visual field of the colored patches had to be relatively small and must be predictable from the segmentation data of the visual cortices. Moreover, the effect of the Chroma condition was also significant in the dorsal pathway (V3A/B), despite the fact that the primary stream for chromatic information processing is known to be the ventral pathway (hV4). Thus, we assumed that brain activity caused by chromatic stimuli did not reflect the strength of chromatic information (in terms of saturation), but rather reflected the strength of achromatic (luminance) information. This means that the patches suppressed the brain activity driven by the achromatic pattern background and that the suppression magnitude was larger under lower-Chroma conditions. Thus, we conducted a control experiment to confirm that the differences in brain activity in the results of the main experiment were not a result of the difference in brain activity driven directly by colored patches. If the results of the main experiment were the results of the activity evoked directly by the patches, the results of the control experiment would present the same pattern as those of the main experiment. Thirteen individuals (nine male and four female, mean age: 23.5 ± 4.3 years) participated in the control experiment and eight of them had already participated in the main experiment.

**FIGURE 5:**
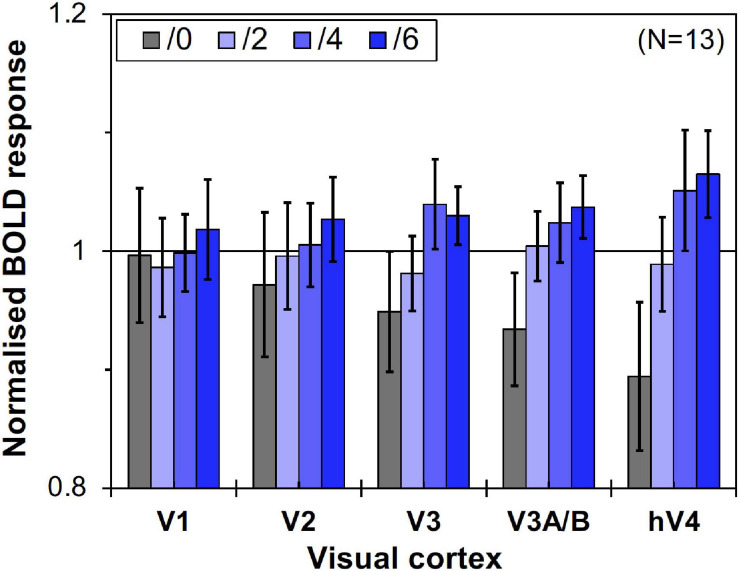
Brain activity of visual cortices under the four Chroma conditions (/0, /2, /4, and /6) in the control experiment. Other notations are the same as in Figure 4.

**FIGURE 6:**
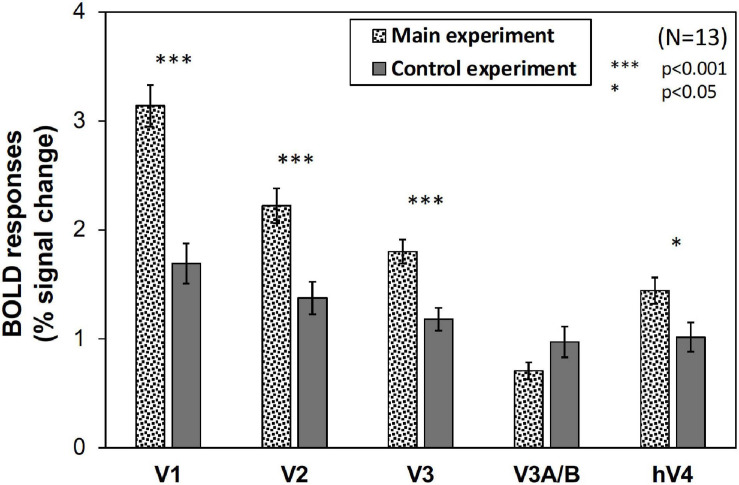
The magnitude (% signal change) of BOLD responses in main and control experiments. The values were averaged across all Chroma conditions and subjects. Error bars denote SEM among the subjects. Significant differences in V1 (*p* < 0.001), V2 (*p* < 0.001), V3 (*p* < 0.001), and hV4 (*p* < 0.05) were confirmed by *t*-test.

However, as can be seen in Figure 5, the results of the control experiment were different from those of the main experiment in terms of dependency on the Chroma value. There were no significant differences among the Chroma conditions (Table 1). The means of the normalized BOLD responses for the Chroma conditions in the control experiment were 1.69% (V1), 1.38% (V2), 1.18% (V3), 0.973% (V3A/B), and 1.02% (hV4). Among the results, brain activity for the patches could not explain the greater depression of the responses under low-Chroma conditions in the background pattern in the main experiment. This lends support to our suppression hypothesis as an explanation of the results of the main experiment. Conversely, the absence of the achromatic pattern background and the simple order of the signal change from Chroma /0 to /6 suggest that the responses measured in the control experiment were evoked by the patches directly, although the differences among Chroma conditions were not statistically significant and the response of V3 could not be explained adequately. Additionally, V1, V2, V3, and hV4 brain activity in the control experiment was significantly smaller in magnitude than that in the main experiment while there is no significant difference in V3A/B (Figure 6).

**FIGURE 7:**
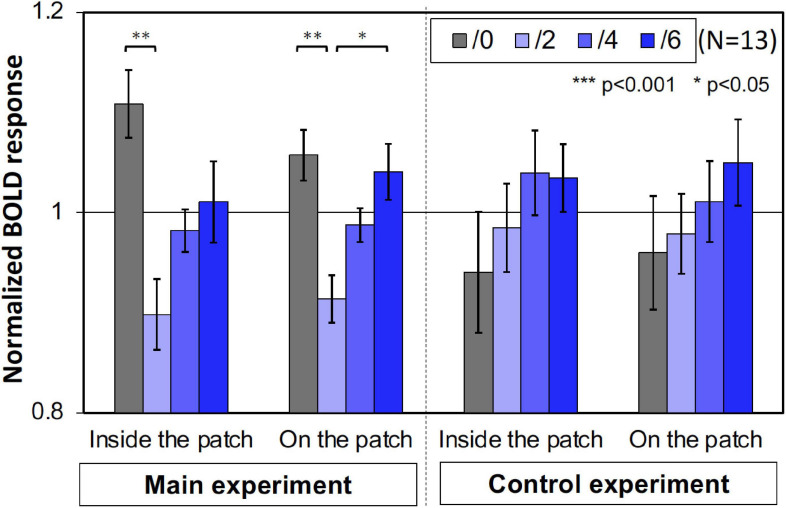
The normalized brain activity of the areas corresponded to inside and on the patches. A one-way ANOVA for the data before normalization, showed a significant effect on both areas only in main experiment (Table 1). Error bars denote SEM. Asterisks denote statistical significance by Tukey’s HSD test (**p < 0.01; *p < 0.05).

**FIGURE 8:**
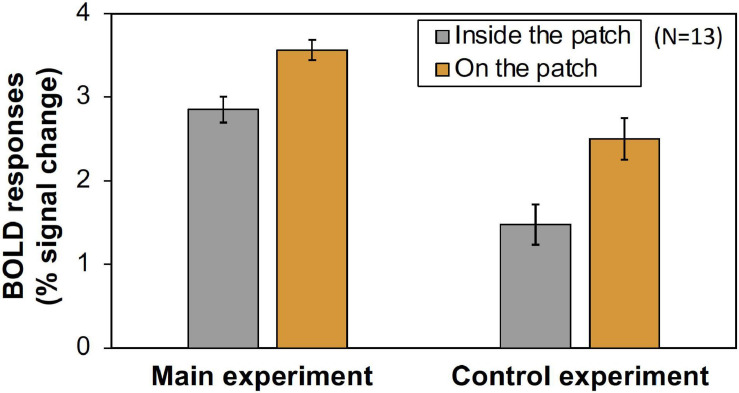
The comparison of the magnitude (% signal change) of V1 BOLD responses inside- and on- the patch in main and control experiments. The values were averaged across all Chroma conditions and subjects. Error bars denote SEM among the subjects. The Inside/On ratio was significantly larger in main experiment (Main experiment: 0.842, Control experiment: 0.609, *p* = 0.011 by *t*-test).

Furthermore, we compared the activities of the areas that retinotopically corresponded to the areas inside the patches (less than 4.5° eccentricity) and on the patches (5.0°–6.5° eccentricity). We used only the V1 BOLD data since the accuracy of the retinotopy in the regions was considered and the magnitude of the BOLD responses in V1 was the maximum (Figure 6). As shown in Figure 7, the activities caused by the Chroma conditions in both areas shared the same common tendency we described in the main experiment (Table 1). This supports our hypothesis that the color patches affected the luminance information. We also confirmed that the rate of the activities inside the patches relative to those on the patch were smaller in the control experiment than in the main experiment (Figure 8), suggesting reasonably good quality in the segmentation of areas inside- and on- the patches.

## Psychophysical Experiment

We conducted a psychophysical experiment to investigate the correlation between the fMRI imaging data and psychophysical data expressing the relationship between color presentation and luminance-dependent perception. The suppression hypothesis was also tested by a psychophysical experiment in which we conducted a luminance contrast discrimination task in order to estimate the strength of the luminance signal when colored patches were presented. Initially we measured perceived luminance contrast in conditions with or without the patches, but we failed to observe significant difference between them because it was difficult for the subjects to respond to luminance contrast of chromatic images. Thus, we employed the slope of fitting function as an index of luminance perception.

**FIGURE 9:**
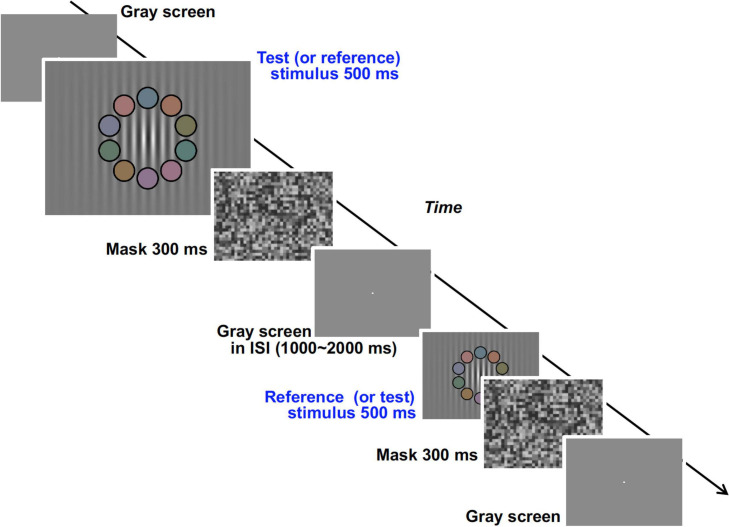
Visual stimuli and time course of the experimental procedure in the psychophysical experiment.

We used an achromatic grating with 10 overlapping colored patches (shown in the second and fifth panels of Figure 9) and measured the response rate of choosing the grating with higher luminance contrast between two gratings presented successively in a temporal two alternative forced choice (2AFC) method (Figure 9). The response rate data as a function of luminance contrast were fitted to a cumulative Gaussian distribution function (psychometric function) and discrimination ability (represented by the deviation of the data distribution) was obtained. From the psychophysical data we obtained the relationship between the Chroma of the patches and the luminance signal which was estimated from discrimination ability.

### Methods

#### Subjects

Eleven subjects (five male and six female, mean age: 24.1 ± 5.4 years) participated in the psychophysical experiment. Six subjects participated in the fMRI experiments and this experiment. Visual acuity and color vision were evaluated in all subjects, as in the fMRI experiments. Note that we excluded the data of one subject because their mean standard deviation under the four Chroma conditions was substantially higher (0.35) than that for other subjects (ranging from 0.05 to 0.12).

#### Visual Stimuli

In the psychophysical experiment, visual stimuli were generated by ViSaGe and presented on a CRT monitor (FlexScan E57T, EIZO) in a dark room (not in the fMRI scanner room). The monitor was calibrated in the same manner as the fMRI experiment. The subjects were fixed at a viewing distance of 54.4 cm by means of a chin rest, and the size of the screen was 31.5° × 23.6°. Though we first tried to use the background pattern in the fMRI experiment, the task was so easy that the slope of their psychometric functions were too steep for comparison between chromatic conditions. Therefore, we decided to employ another visual stimulus in the psychophysical experiment rather than maintaining consistency of visual stimuli appearance between the two experiments (see section “Differences of Visual Stimuli Between the Experiments” for details). In the temporal 2AFC method two types of visual stimuli, test and reference stimuli, were presented sequentially in random order (Figure 9). The stimuli consisted of an achromatic grating (0.67 cycles per degree) enveloped by a decremental exponential-function (3.75° for 1/e decline) and 10 colored patches with a black fringe overlapping the grating (Supplementary Figure 2). The Gabor function was not used for the envelope of the grating because the decline rate of the peak luminance produced by the Gaussian envelope was too high and the gratings at the low luminance contrast part (ex. 2.5%) were overlapping with some of the patches (as can be seen in Supplementary Figure 2). The number of visible grating peaks could have influenced subject judgment of luminance contrast. The mean luminance of the grating and the luminance of the patches were set to 19.8 cd/m^2^ in both test and reference stimuli. The direction and phase of the stripes in the grating were also fixed. In the reference stimuli, the spatially-maximum Michelson contrast (negative contrast at the center) of the luminance grating was fixed at 50%. In the test stimuli, the maximum contrast was varied from 34 to 66%. We also manipulated the Chroma of the patches (from /0 to /6) and other details of the patches (size, position, arrangement of colors, and color properties) to be the same as those in the fMRI experiments. The Chroma condition of the test and the reference stimuli were the same in the trial but were changed pseudorandomly between trials. The mask pattern in the psychophysical experiment consisted of achromatic squares (1.5° × 1.5°) of which the luminance was chosen randomly from among eight levels ranging from 0.32 to 30.37 cd/m^2^ and the mean luminance was 15.38 cd/m^2^.

#### Procedure

In the trial, test and reference stimuli were presented sequentially in random order (Figure 9). The duration of both stimuli was 500 ms, and a mask stimulus was presented for 300 ms after each stimulus. ISI between test and reference stimuli was randomly set between 1,000 and 2,000 ms. After the appearance of the two stimuli, the subject reported which stimulus had higher achromatic (luminance) contrast in the grating. Except during the time when the test, reference, and mask stimuli were being presented, a bright fixation point was presented on a uniformly gray background (19.8 cd/m^2^) (Figure 9). There were 36 conditions in total (nine conditions for contrast of the test stimuli by four chromatic conditions) and all conditions were presented in each session. Each subject performed 12 experimental sessions. Throughout the experiment the subjects received no feedback about their responses, and were not informed which (first or second) stimulus was the test stimulus in each trial. The data for the initial two sessions were discarded so as to avoid the strong learning effect in the earliest sessions.

#### Data Analysis

The data was fit with a cumulative Gaussian distribution function (GDF) as the psychometric function (Eq. 3).

(3) $$P\,\left(x\right)=\int_{-\infty }^{x}\frac{1}{\sqrt{2\,\pi \,\sigma^{2}}}\,\text{exp}\,\left(\frac{-{\left(x-\mu \right)}^{2}}{2\,\sigma^{2}}\right)\,\text{d}\,x$$

**FIGURE 10:**
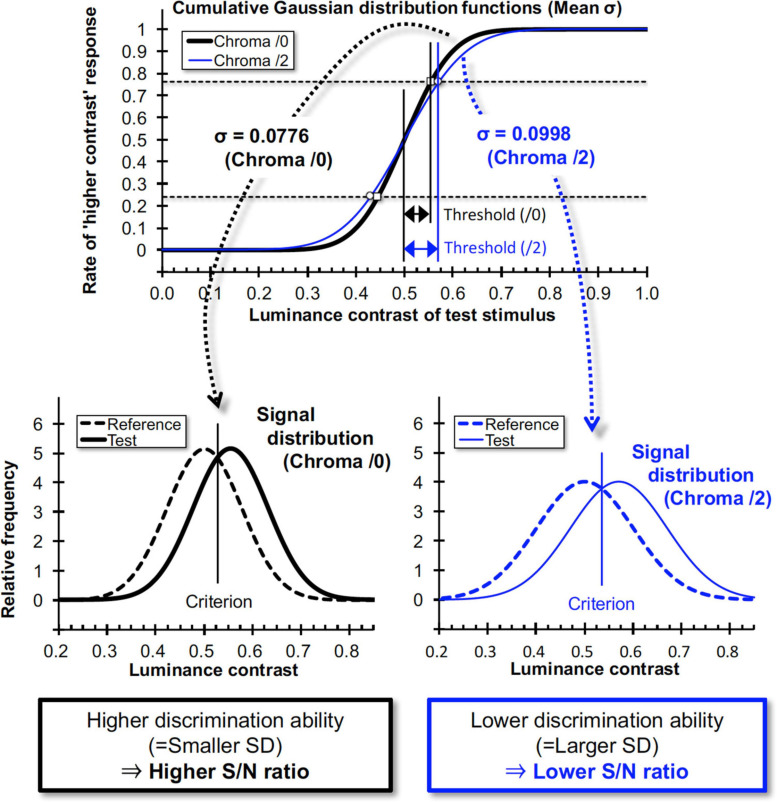
Relationship between cumulative Gaussian distribution functions for data fits and signal distributions in signal detection theory. When the slope of the “higher contrast” response rate is high, the neural Signal/Noise ratio will be high, and vice versa. ***σ*** values here are means of all subjects for each Chroma value.

Where ***x*** is the maximum luminance contrast (Michelson contrast) of the grating in the test stimulus, **μ** is the contrast at 50% probability (chance level), and ***σ*** is the standard deviation of the distribution function. In fits to the probability data using this function, the optimized parameters **μ** (mean of the data) and ***σ*** were obtained. A larger ***σ*** results in a gentler slope of the cumulative GDF and indicates higher contrast discrimination threshold at defined criterion (i.e., 75%) as shown in Figure 10. In signal detection theory this larger ***σ*** gives rise to lower and wider signal distribution functions and corresponds to a lower S/N ratio of the discrimination task (Figure 10). Under the constant noise assumption the lower S/N ratio indicates lower signal strength for the discrimination task. Thus, we employed ***σ*** values as an index for luminance contrast sensitivity.

### Results

**FIGURE 11:**
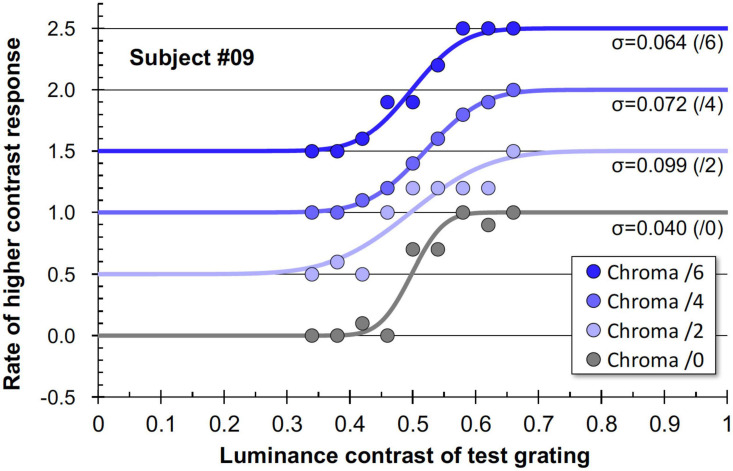
Example data and psychometric functions for one subject under four Chroma conditions. The ordinate was defined as the data for Chroma /0 condition. Other data were vertically shifted in multiples of 0.5 to clarify. The standard deviations (***σ***) for all conditions are shown in the panel.

**FIGURE 12:**
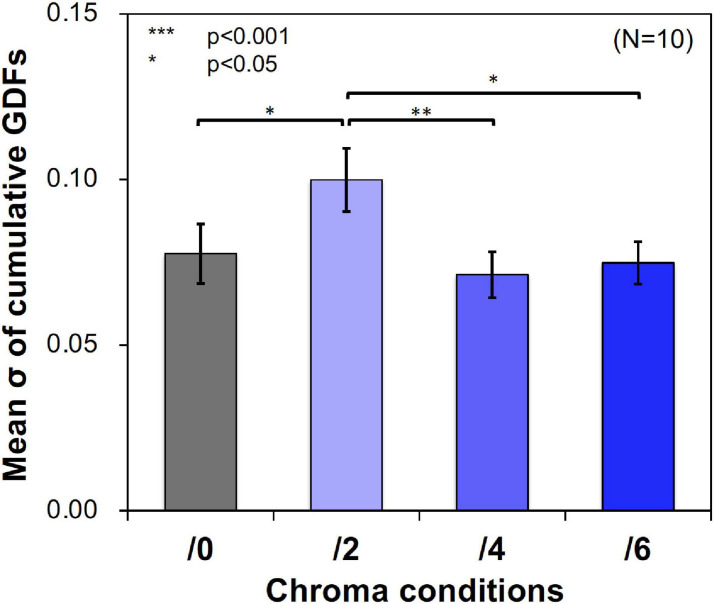
Mean standard deviation (***σ***) of data distributions. Standard deviations were calculated using cumulative Gaussian distribution functions (GDFs) fitted to response rate data of luminance contrast discrimination under the four Chroma conditions (/0, /2, /4, and /6) in the psychophysical experiment. Error bars denote SEM. Asterisks denote statistical significance by Tukey’s HSD test (**p < 0.01; *p < 0.05).

Figure 11 shows examples of the data and psychometric functions for one subject in the four Chroma conditions. Figure 12 shows the mean of the standard deviation, ***σ*** under the four Chroma conditions. A one-way repeated measures (within subjects) ANOVA showed a significant main effect of Chroma condition (Table 2), and the standard deviation under the Chroma /2 condition was significantly larger than those under the other Chroma conditions (by Tukey’s HSD test: Chroma /0: *p* = 0.046, Chroma /6: *p* = 0.021, and Chroma /4: *p* = 0.007). This result indicates that luminance contrast discrimination ability was worse in the Chroma /2 condition, and this result is consistent with the result of fMRI experiment that the suppression of luminance information was most prominent in Chroma /2 patches.

**TABLE 2:** ANOVA results of psychophysical experiment.

**Source**	**D*f***	**Sum Sq**	**Mean Sq**	***F* value**	***p***
Chroma condition	3	0.005	0.002	5.180	0.006**
Subject (DV)	9	0.015	0.002		
Residuals	27	0.009	0.000		

**p < 0.01.

## General Discussion

The relationship between luminance, mostly treated in a form defined by luminance in previous studies, and color has been studied extensively in various visual stimuli (Moutoussis, 2015). The results of previous psychophysical studies using gratings show that presentation of color contrast has a masking effect on luminance contrast and vice versa (Mullen and Losada, 1994; Moutoussis, 2015). Switkes et al. demonstrated that presentation of luminance information (i.e., mask grating) suppressed responses mediating chromatic information (i.e., test grating), and that suppression is stronger at a certain luminance contrast (about 2%) than for higher or lower contrasts. However, their report does not indicate the suppression of luminance information by chromatic information at low color contrast; rather, the results show monotonic increment of suppression (i.e., higher threshold) as the color contrast increased, indicating a simple masking effect (Switkes et al., 1988).

**FIGURE 13:**
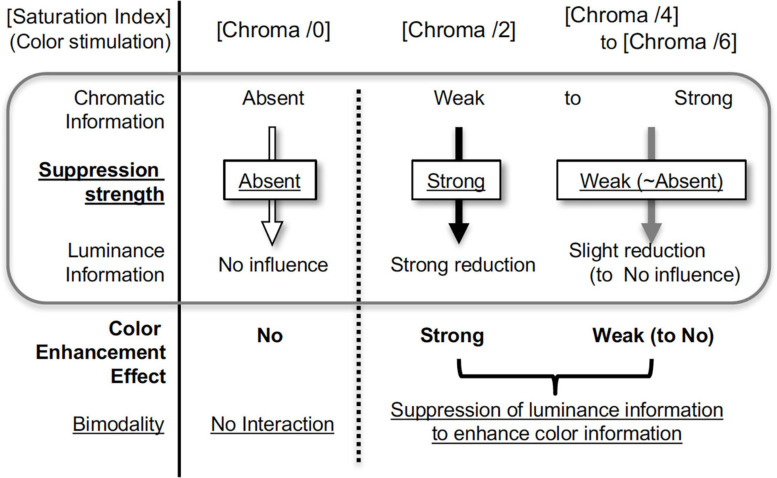
Scheme of color enhancement model.

Here, we first demonstrated the suppression of brain activity by the presentation of chromatic stimuli. We also demonstrated suppression due to dependency of saturation on stimulus colors in both brain activity and luminance contrast sensitivity; the suppression of the luminance information mostly occurred with the presentation of color with low saturation (low Chroma), and little or no suppression was observed with the presentation of no color (Chroma /0) or highly-saturated colors (Chroma /6) (Figure 13). We note that most of previous fMRI studies of color vision focused on responses mediating chromatic information in visual objects. In our study an achromatic background was dominant in the visual stimuli and we succeed in identifying the effect of color presentation on brain activity through examination of responses evoked by achromatic stimulation.

### Luminance Suppression and Color Enhancement Model

Brain activity relating to luminance information was only suppressed when the colored patches were attached to an achromatic background, and the amount of suppression did not simply correspond to the color strength (Chroma), recognized perceptually as intensity of chromatic information. Instead, suppression occurred only when the visual stimuli contained low saturation (low chromatic information), and did not occur when color (chromaticness) of visual stimuli was not recognized or when chromaticness of stimuli was sufficiently strong. These suppression-related phenomena can be accounted for by the hypothesis of the color enhancement model using bimodality; if the strength of the chromatic information is relatively weak, the chromatic information will be relatively enhanced to achieve a better balance between chromatic and luminance information (Figure 13). In terms of benefit, weak colors are perceived more clearly under suppression, although the S/N ratio of luminance information deteriorates as a result.

In the psychophysical experiment, the signal strength of luminance information was measured indirectly from the rate of the “higher contrast” response curve with signal detection theory (details in section “Methods” and Figure 10). Under the assumption that the noise level for one psychophysical task is almost constant within one subject, the lower S/N ratio indicates that lower signal strength was used for the discrimination task. The results of the psychophysical experiment also indicate that the S/N ratio of luminance information was lower in the Chroma /2 condition than in other conditions. Conversely, without the achromatic background, averaged brain activity tends to correspond to the Chroma value, especially in hV4. Human visual cortices contain color selective cells, and the activity of those cells corresponds to the strength of chromatic information (the Chroma value in our experiments). Since hV4 contains a substantial number of those cells and strongly responds to chromatic visual stimuli (Livingstone and Hubel, 1988; Zeki et al., 1991; Bartels and Zeki, 2000; Wade et al., 2008; Brouwer and Heeger, 2009), it is likely that this result indicates activity of color preference cells.

### Mechanism

Chromatic information is mainly processed in the ventral and lateral visual pathways (Wade et al., 2008; Hansen and Gegenfurtner, 2017), although some reports indicate that chromatic information is also utilized in the dorsal pathway (Tootell et al., 1995; Wandell et al., 1999; Takemura et al., 2016). The suppression occurred over the primary, dorsal, ventral, and lateral visual pathways. Thus, it is expected that suppression initially occurs in the early stage of the visual information process and will be propagated to higher stages not as a chromatic signal but as a luminance signal. Our results suggest that suppression occurred latest in V1. This corresponds to the results of a recent study using both Visual Evoked Potential (VEP) measurement and psychophysical tests, which indicate that the inhibitory signal for brightness and color interaction arises in a recurrent inhibitory network in V1 (Xing et al., 2015); cells responding to both luminance and color stimuli in macaque V1 have been found (Livingstone and Hubel, 1984; Johnson et al., 2001). Thus, one possible explanation for the suppression mechanism is that these cells respond to luminance during observation of achromatic stimuli, but respond to color during observation of chromatic stimuli (particularly when the color signal is weak) causing a reduction of aggregate luminance response. Another possible explanation is that chromatic information directly suppresses the response of luminance selectivity cells. In either case, there must be a determinant signal for suppression in V1 and/or higher visual cortices.

We manipulated the Munsell Chroma value of the patches and controlled the saturation of presented colors as the variable. However, the mechanism determining the strength of the suppression is still not clear. A neuron corresponding to a particular value of saturation has not been found, at least in early visual cortices; it has been considered that chromatic information, including saturation (strength of color), is coded by the input from single- and/or double- opponent cells consisting of L - M and S - (L + M) chromatically opponent systems in V1 (Lee, 2011; Shapley and Hawken, 2011). Moreover, brain activity patterns were different among hues of stimuli (Engel et al., 1997; Mullen et al., 2007; Kuriki et al., 2015). Thus, the presence and strength of suppression might be the result of computational processes for saturation using the responses of those hue-processing systems.

The suppression could also be accounted by the change of attentional states when the difference between the fMRI and psychophysical experiments is considered. In our fMRI experiments the subjects were not required to attend to some specific feature in their field of vision actively. However, the colored patches in our experiments might raise bottom-up attention that modulated the BOLD responses in visual cortices. Thus, the attention effect could also have a role to attenuate the luminance signal relative to the chromatic signal, although it is hard to explain how this attenuation would be dominant only at low saturation (Chroma /2) at which the color of the patches were weak and when the subjects were not asked to observe nor to perform any tasks on these color patches. The results of a previous study (Switkes et al., 1988) suggest that the interaction between luminance and chromatic information, which was observed as a change of thresholds, occurred when active attention was not required. It may be a possible explanation is that the patches of the low saturated colors did not evoke a strong attention effect causing the interaction between luminance and chromatic information to be observed strongly in this condition, but the patches at higher saturated colors evoked strong attention effect that caused the interaction not to be observed. This hypothesis can correspond to the psychophysical results (Figure 12), however, it is still difficult to explain the fMRI data showing the gradual change of the suppression (Figure 4). In the case of the psychophysical experiment, the subjects had to judge the higher luminance contrast in a temporal two alternative forced choice task and were required to pay attention to the grating actively. It might indicate that color patches captured some amount of attentional resources from the grating unconsciously and the suppression might be reduced in the two higher saturation conditions.

### Differences of Visual Stimuli Between the Experiments

Different visual stimulus patterns were used in the fMRI and psychophysical experiments because of the purpose of the experiments. In the fMRI experiment, the background pattern consisted of gray ellipses to stimulate neurons in the visual cortices that are tuned to multiple orientations and spatial frequencies. On the other hand, in the psychophysical experiment the vertical gratings with color patches were compared in a 2AFC method to select the higher luminance contrast. There were two reasons to employ different visual stimuli in the psychophysical experiment in exchange for consistency of appearance between experiments.

The first reason was that we thought the psychometric functions we obtained with the ellipse background pattern used in the fMRI experiment were too steep due to the existence of many luminance edges. The subjects could detect the contrast easily by simply attending to the edge of the highest contrast. Thus, the contrast at the sharp luminance edge had to be minimized to obtain a less steep psychometric function. Additionally, attending to a certain luminance edge may induce a difference in attentional state between the fMRI experiment and the psychophysical experiment. We therefore tried to design the experiment in such a way that the subject would not to pay attention to only a certain luminance edge of the visual stimulus.

The other reason was that in the psychophysical experiment, we tried to measure the luminance contrast sensitivity using psychometric functions in order to obtain the magnitude of the luminance signal. Although a psychometric function can be obtained by a complex visual stimulus (Ueda et al., 2018; Li et al., 2021), in our case we were afraid we would not be able to obtain the magnitude of the luminance signal from the function. Therefore, we thought it would be better to make the visual stimulus simple because we did not have a specific model to estimate the magnitude of the luminance signal from the psychometric functions obtained from the visual stimulus with this ellipse background pattern. For example, we were afraid of possible interactions in the contrast detection among the multiple orientations and spatial frequencies and that this ellipse background pattern would make the results unpredictable. Thus, we employed the grating pattern to minimize these interactions. Therefore, we can consider the ***σ*** values (Eq. 3) as the index for luminance contrast sensitivity of one orientation and one spatial frequency, and they can be used to predict the magnitude of the luminance signal. We consider the results obtained by the fMRI and psychophysical experiments to reflect the magnitude of the luminance responses between chromatic conditions; however, these results might be influenced by additional phenomena due to the differences in the visual stimuli.

### Arguments

It is also possible to explain the psychophysical data of this study by the gamut expansion effect (Brown and MacLeod, 1997), in which strength of color is expanded or compressed from zero (neutral color) to the most saturated color in the visual stimulus. However, the gamut expansion effect on color perception can also be accounted by the aforementioned suppression hypothesis. When visual stimuli include highly saturated (high-Chroma) colors, suppression, i.e., the enhancement of perceived saturation, is weak or absent, so that perceived saturation is almost the same as presented saturation. On the other hand, when the visual stimuli are composed of desaturated (low-Chroma) colors, suppression works strongly and perceived saturation is higher than presented saturation.

Kim and Mullen (2016) reported that humans tend to use the chromatic edge as well as the luminance edge to recognize natural scenes, and they suggest there is a weak masking effect of luminance by color, and the results of our experiments also suggests that chromatic information suppresses luminance information.

The results of our psychophysical experiment were consistent with the results of previous studies in which the effect of chromatic information on luminance contrast sensitivity was investigated (Miquilini et al., 2017; Sousa et al., 2020) in that the presence of color reduces luminance contrast sensitivity. Nevertheless, the correlative relationship between contrast sensitivity and saturation of color was the opposite in our result. It can be explained by two differences between our experiments and theirs. The first is the way to measure contrast sensitivity; the detection threshold of contrast was measured in the previous studies, while our experiment measured contrast discrimination performance above the detection threshold. The second is the spatial arrangement of the visual stimuli; color and luminance information were spatially overlapped in the previous studies and separated in our experiment. Thus, we could expect that the effect of chromatic information to luminance contrast measured in our experiments depends complexly on color appearance, which may involve a possible attention effect, whereas the strength of the chromatic signal could be a dominant factor in the results of previous studies.

There are some limitations of this investigation of suppression. Firstly, our experiment couldn’t reveal the hue factor of suppression, especially in terms of cone types mediating hues (Shinomori and Werner, 2012; DeLawyer et al., 2018), because the patches in our visual stimuli were distributed across hues. However, psychophysical data show that suppression occurs for all hues and the tendencies are the same across all hues (Kingdom et al., 2010). Secondly, when the Chroma value of the patches was decreased (lower than /2 and below a certain Chroma threshold) it seemed that suppression disappeared or decreased. The mechanism and behavior of suppression around the threshold point is still unclear, so detailed experimentation is essential to pursue these questions. Furthermore, investigation is needed regarding possible spatial structure, such as an effective spatial range of the suppression, since it is reasonable to expect suppression only in the region around the color stimulus (Xing et al., 2015) to avoid decreasing luminance sensitivity over the entire visual field. If such a range exists its precise spatial structure should be determined because that spatial structure could be of use in investigations of the suppression mechanism.

## Conclusion

The human visual system generally processes chromatic and luminance signals separately. Nevertheless, interaction between the two signal types have been suggested. We performed fMRI measurements of brain activity during exposure to visual stimuli consisting of chromatic and luminance components. Brain activity driven by luminance components was suppressed by chromatic components and the suppression was stronger when the presented chromatic stimuli were less saturated, although suppression was absent when there was no chromatic component. The psychophysical measurements of luminance contrast discrimination also support luminance signal reduction. These results directly imply that the interaction enhances chromatic information by supporting the use of weak color among visual stimuli, and explain the phenomenon that weaker-color images appear to have lower achromatic contrast.

## Data Availability Statement

The raw data supporting the conclusions of this article will be made available by the authors, without undue reservation.

## Ethics Statement

The studies involving human participants were reviewed and approved by Kochi University of Technology Research Ethics Committee. The patients/participants provided their written informed consent to participate in this study. Written informed consent was obtained from the individual(s) for the publication of any potentially identifiable images or data included in this article.

## Author Contributions

IN and KS designed and carried out the experiments, analyzed the data, and wrote the manuscript. Both authors contributed to the article and approved the submitted version.

## Conflict of Interest

The authors declare that the research was conducted in the absence of any commercial or financial relationships that could be construed as a potential conflict of interest.
