# Supplementary material for: Suppression of Luminance Contrast Sensitivity by Weak Color Presentation
Source: Front Neurosci. 2021 Jun 28;15:668116. doi: 10.3389/fnins.2021.668116 (PMC8273178; doi:10.3389/fnins.2021.668116)
Supplement: Supplementary file 1 [file Data_Sheet_1.docx]

Supplementary Material

# Supplementary Figures


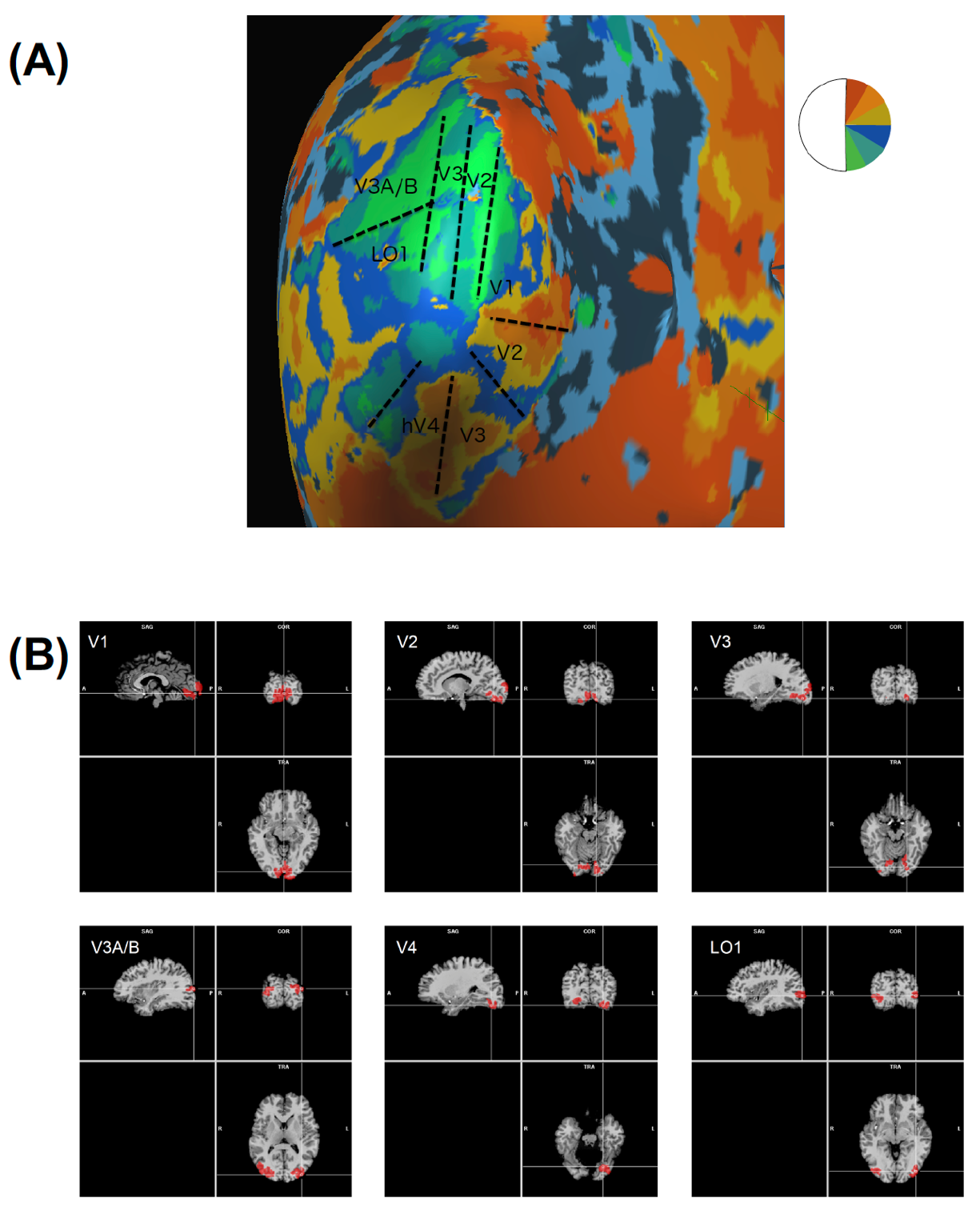


**Supplementary Figure 1.** Example of visual cortex segmentation. Segmentation of the cortex surface (A) and segmentation between white and gray matter (B). The top right circle in panel (A) denotes the color-codes corresponding to the field positions in visual stimulation and the responses at ROIs.


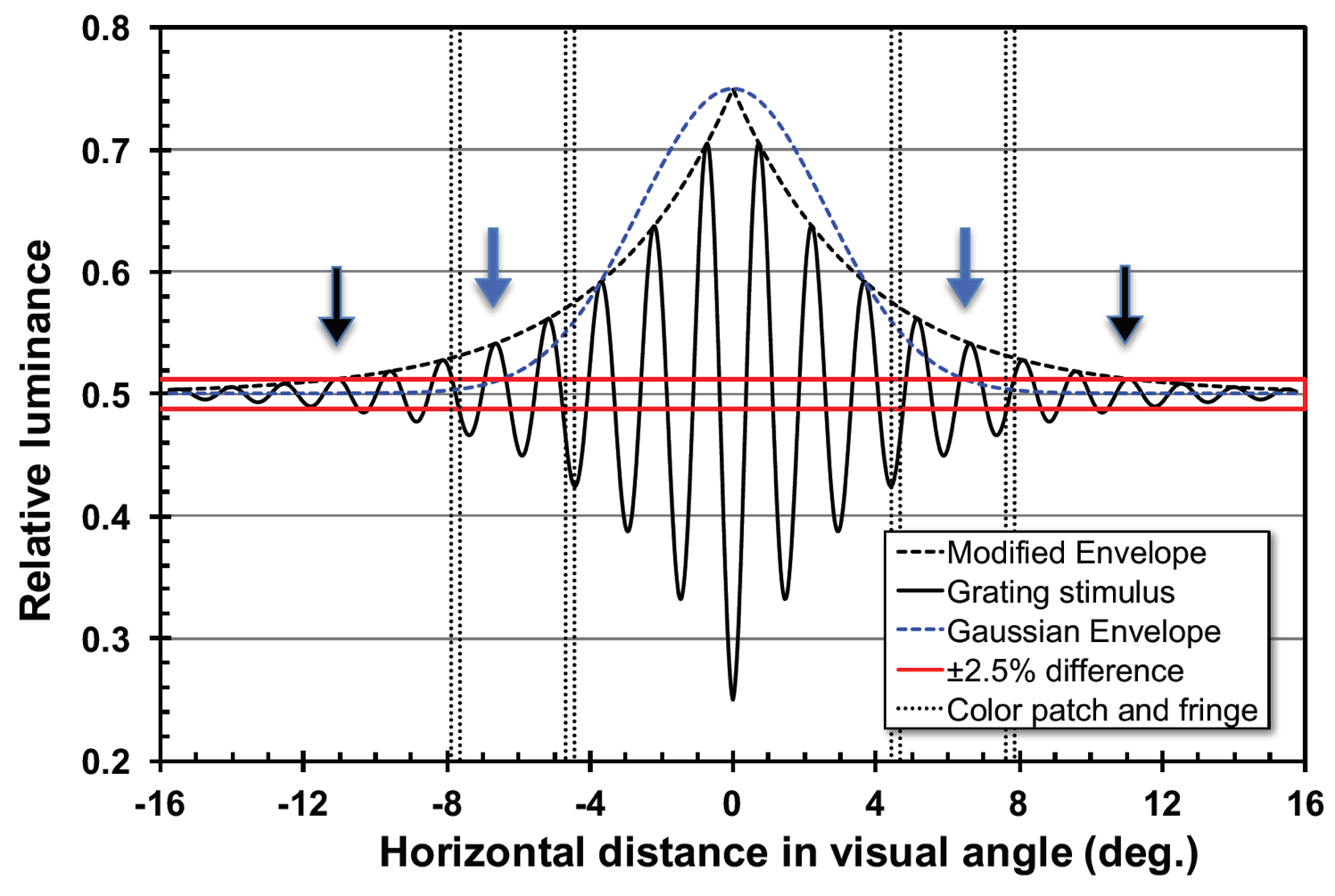


**Supplementary Figure 2.** Luminance profile of the grating stimulus in psychophysical experiment. The grating stimulus (black solid curve) was the minus cosine function in the horizontal direction, enveloped by a decremental exponential-function (as a function of distance from the center). Luminance profile is at the vertical center in the case of a 50% luminance contrast. Black and blue dotted curves denote the exponential envelope (decremental exponential-function) and Gaussian envelope, respectively. Red horizontal lines denote a ± 2.5% difference from the background luminance. Black and blue arrows indicate the first peaks from the center that are less than 2.5% increments from the mean luminance in the exponential and Gaussian envelopes, respectively. Vertical dotted lines denote the theoretical position of chromatic patches and fringes, although the centers of the colored patch and the fringe were not placed exactly at the vertical center because all patches rotated around the center.
